# Letter from G. P. Putnam’s Sons

**Published:** 1882-03

**Authors:** 

**Affiliations:** New York


### Domestic Correspondence.

### ARTICLE IX.

NEW YORK, FEBRUARY 3, 1882.

EDITORS MEDICAL JOURNAL AND EXAMINER:—The assertion having been made in various quarters that the *Journal of Nervous and Mental Diseases*, heretofore edited by Dr. J. S. Jewell, of Chicago, and of which for the past five years we have been the publishers, has been "sold out" or discontinued, we desire to state to the medical profession that there are no grounds for any such statement. There has been no break in its publication, no intention to discontinue it, and no announcement of any such intention. Dr. J. S. Jewell having been compelled on the ground of ill health to resign the editorial management, has transferred the same to Dr. W. J. Morton, of this city. He continues, however, as an associate editor, to give to the *Journal* the service of his long professsonal and editorial experience. In addition to Dr. Jewell, the editor has secured the coöperation, as associate editors, of Drs. Seguin, Hammond, Bannister, Clymer and Ott, and the *Journal* gives every promise, under its present able management, of widely extending its work and influence.

By giving this note a place in your pages, you will oblige,

Yours respectfully, G. P. PUTNAM'S SONS.

